# Bothersome Hot Flashes Following Neoadjuvant Androgen Deprivation Therapy and Stereotactic Body Radiotherapy for Localized Prostate Cancer

**DOI:** 10.7759/cureus.55729

**Published:** 2024-03-07

**Authors:** Sarthak Shah, Abigail Pepin, Simran Jatar, Jessica Hsueh, Lindsey Gallagher, Malika T Danner, Alan Zwart, Marilyn Ayoob, Thomas M Yung, Deepak Kumar, Nima Aghdam, Paul D Leger, Nancy A Dawson, Suy Simeng, Sean P Collins

**Affiliations:** 1 Radiation Medicine, MedStar Georgetown University Hospital, Washington, USA; 2 Radiation Oncology, University of Pennsylvania Abramson Cancer Center, Philadelphia, USA; 3 Medicine, Biotechnology Research Institute, North Carolina Central University, Durham, USA; 4 Radiation Oncology, Beth Israel Deaconess Medical Center, Harvard Medical School, Boston, USA; 5 Oncology, MedStar Georgetown University Hospital, Washington, USA

**Keywords:** prostate cancer, sbrt (stereotactic body radiotherapy), cyberknife, adt (androgen deprivation therapy), hot flashes

## Abstract

Background: Androgen deprivation therapy (ADT) improves local cancer control in unfavorable localized prostate cancer treated with radiotherapy. ADT is known to cause hormonally related symptoms that resolve with testosterone recovery. Hot flashes are particularly burdensome. This study sought to evaluate the timeline of hot flashes following short-course ADT and stereotactic body radiotherapy (SBRT) as well as its relationship with testosterone recovery.

Methods: Institutional IRB approval was obtained for this retrospective review of prospectively collected data (IRB#: 2009-510). ADT was initiated three months prior to the start of SBRT. Hot flashes were self-reported via question 13a of the Expanded Prostate Index Composite (EPIC)-26 prior to ADT initiation, the first day of robotic SBRT, and at each follow-up (one, three, six, nine, 12, 18, 24, and 36 months). The responses were grouped into three relevant categories (no problem, very small-small problem, and moderate-big problem). Scores were transformed to a 0-100 scale with higher scores reflecting less bother. Testosterone levels were measured at each follow-up.

Results: From 2007 to 2010, 122 localized prostate cancer patients (nine low-, 64 intermediate-, and 49 high-risk according to the D’Amico classification) at a median age of 72 years (range 54.5-88.3) were treated with short course ADT (three to six months) and SBRT (35-36.25 Gy) at Georgetown University Hospital. Thirty-two percent were Black and 27% were obese. Seventy-seven percent of patients received three months of ADT. At baseline, 2% of men experienced hot flashes that were a “moderate to big problem” and that proportion peaked at the start of SBRT (45%) before returning to baseline (2%) nine months post-SBRT with a cumulative incidence of 52.4%. The median baseline EPIC-26 hot flash score of 94 declined to 50 at the start of SBRT but this returned to baseline (92) by six months post SBRT. These changes were both statistically and clinically significant (MID = 9.5083, p<0.01). Testosterone recovery (> 230 ng/dL) occurred in approximately 70% of patients by 12 months post SBRT. Resolution of hot flashes correlated with testosterone recovery.

Conclusion: Bothersome hot flashes occur in greater than 50% of men treated with neoadjuvant ADT. Resolution of hot flashes occurs in the majority of patients within one year after treatment. Reassurance of the temporary nature of hot flashes may assist in reducing patient anxiety. Measuring testosterone levels at follow-up visits may allow for anticipatory counseling that may limit the associated bother.

## Introduction

The current treatment paradigm for unfavorable and high-risk prostate cancer involves a combination of androgen deprivation therapy (ADT) in combination with radiotherapy [1-3]. The addition of ADT to radiotherapy improves prostate cancer-specific mortality and distant metastasis rates in men with unfavorable- and high-risk prostate cancer [4,5]. Despite this, ADT is associated with impaired quality of life and detriments related to adverse side effects. In particular, low testosterone from androgen suppression is associated with hypogonadal symptoms such as fatigue, depression, gynecomastia, weight gain, and hot flashes [1,6].

Hot flashes are one of the most bothersome toxicities to men following ADT, but hot flashes are generally benign findings that self-resolve [7]. Hot flashes can also be treated with a number of pharmacologic agents including gabapentin, Megace, and venlafaxine. About 70% to 80% of men will experience hot flashes while undergoing ADT [8,9]. Due to symptomatic bother, hot flashes may decrease adherence to ADT recommendations [10]. Patients reported quality of life scores often improve to baseline in the months following ADT corresponding to testosterone recovery (TR).

The timeline to TR following androgen deprivation is variable. Previous studies have defined TR as a return to >230 ng/ml [11]. The time to TR is variable and dependent on patient and treatment characteristics including age, race, BMI, and duration of ADT [12,13]. Older and obese men have a shorter time to TR, while black men have a faster time to TR [12,14,15]. The longer duration of ADT correlates with a slower trajectory to TR [12]. We have previously reported that 71% of patients recovered to a eugonadal state around 12 months, with a mean recovery time of four months post-stereotactic body radiotherapy (SBRT) [16]. This study sought to understand the onset and resolution of hot flashes, as well as evaluate the relationship between TR and hot flash onset and resolution.

This article was previously presented as a poster at the 2023 ASTRO Annual Meeting on October 2, 2023.

## Materials and methods

Patient selection

Patients eligible for inclusion in this study had histologically confirmed localized prostate cancer treated with a combination of short-course ADT and SBRT and had a minimum of three years of follow-up. Approval from the MedStar Georgetown University Institutional Review Board was obtained for a retrospective review of prospectively collected data in our institutional database (IRB#: 2009-510).

Treatment planning and delivery

All patients received between three to six months of ADT consisting of the luteinizing hormone-releasing hormone agonist, leuprolide (22.5 mg). In general, SBRT was delivered three months after the first injection to maximize prostate size reduction and minimize radiation dose to surrounding healthy tissues [17]. SBRT was delivered utilizing the CyberKnife robotic radiosurgical system (Accuray, Inc., Sunnyvale, CA, USA). Required fiducial placement, treatment planning magnetic resonance imaging (MRI), and computed tomography (CT) simulation procedures have been previously described [18,19]. The clinical target volume (CTV) was defined as the prostate and proximal seminal vesicles. The CTV was expanded 5 mm in all directions except 3 mm posteriorly to generate the planning target volume (PTV). Patients were treated to a prescription dose was 35-36.25 Gy to the PTV delivered in five fractions over two weeks based on treatment planning performed using Multiplan (Accuray Inc., Sunnyvale, CA, USA). Treatment beams that directly traversed the testis were blocked and scatter dose was kept to a minimum [17]. In general, patients began treatment between two and and weeks after the treatment planning scans.

Follow-up and statistical analysis

Serum testosterone levels were obtained before the first SBRT treatment and during routine follow-up visits every three months for the first year and every six months thereafter. In general, serum samples were collected in the morning to limit the impact of circadian variance [17]. TR was defined as reaching a serum testosterone level of at least 230 ng/mL. Hot flash bother was assessed using the Expanded Prostate Index Composite (EPIC)-26: Question 13a prior to ADT initiation, the first day of SBRT, and at each follow-up. The EPIC questionnaire asks patients to assess both sexual and hormonal function, and question 13a specifically assesses hot flashes at these time points.

To statistically compare changes in EPIC question scores at each time point, the level of responses was assigned a score and transformed onto a 0-100 scale with lower scores reflecting worsening sexual or hormonal symptoms [19]. The minimally important difference (MID=9.5) utilized for the question 13a response was determined by half plus or minus the standard deviation from baseline values [20,21].

## Results

One hundred twenty-two patients with localized prostate cancer treated with short-course ADT and prostate SBRT at Georgetown University Hospital from 2013 to 2019 were included in this analysis. Their baseline characteristics are summarized in Table 1. Our patients were ethnically diverse with a median age of 72 years (range 54.5-88.3). Thirty-two percent were black and 27% were obese. The patients had high levels of comorbidities with 52% of patients having a Charlson-Comorbidity Index of one or more. Using the D’Amico risk classification, nine patients were low-, 64 intermediate-, and 49 high-risk. Approximately 77% of patients received three months of ADT.

**Table 1:** Patient characteristics and treatment SBRT - stereotactic body radiotherapy; AUA - American Urologic Association

	Percent of Patients
	(n=122)
Age (years): Median 77 (55–88)	
60–69	6.40%
70–79	40%
>80	53%
Race	
White	52%
Black	32%
Other	16%
Prostate Volume (cc)	Median 41.1 (7 - 160)
Body Mass Index (kg/m²)	
<18.5	1%
18.5–24.9	23%
25.0–29.9	48%
30.0–34.9	20%
35.0–39.9	4%
40.0–44.9	3%
Charlson Comorbidity Index	
0	48%
1	25%
2	16%
3	11%
Risk Group (D’Amico)	
Low	7%
Intermediate	52%
High	40%
Hormone Therapy	
3 months	77%
4 months	8%
6 months	15%
SBRT Dose	
35	34%
36.25	66%
AUA Baseline	
0–7 (Mild)	39%
8–19 (Moderate)	45%
≥20 (Severe)	16%

The prevalence of hot flashes prior to and after SBRT treatment is shown in Table 2. At the time of initial consult, 90% of patients reported no problem with their hot flashes. Only 7% endorsed a very small/small problem, and 2% endorsed a moderate/big problem with hot flashes. At time of ADT, 73% of patients reported hot flashes; 28% described hot flashes as very small or a small problem and 45% as a moderate to big problem. Scores of patient-reported hot flashes decreased significantly following treatment. The bother of hot flashes (20%) remained clinically and statistically significant until the six-month time point (MID = (9.5, p<0.01), but after this time point, hot flash bother returned to baseline levels.

**Table 2:** Hot flashes bother following short course ADT and SBRT by time point ADT - androgen deprivation therapy; SBRT - stereotactic body radiotherapy

	Initial Consult	Start Pre-Treatment	1 month	3 months	6 months	9 months	12 months	18 months	24 months	36 months
No problem (%)	90	27	29	53	78	88	89	93	91	90
Very Small/Small Problem (%)	7	28	33	29	14	10	9	7	6	7
Moderate/Big Problem (%)	2	45	38	18	8	2	1	0	2	2
Patient Response (N)	94	122	112	107	101	84	75	69	84	94
P-value		>.01	>.01	>.01	>.01	.52	.58	.34	.93	1

The hot flash mean reported EPIC scores can be seen in Table 3. Hot flashes were not bothersome at baseline, with the mean score being 94.3. Hot flash bother increased to 50.9 at time of radiation start. Following SBRT, scores increased gradually to 55 and 73 at one and three months post-SBRT, respectively, and returned to baseline levels by 12 months (p=0.58). The hot flash mean scores remained stable at this level for the three-year follow-up period, eventually reaching 97 at 36 months (Table 3).

**Table 3:** Hot flash mean reported score following short course ADT and SBRT for prostate cancer ADT - androgen deprivation therapy; SBRT - stereotactic body radiotherapy

Time	Score	Confidence Interval (95%)
Baseline Score	94.3	90.5-98.1
SBRT start	50.9	47.1-54.7
1 month	55.4	51.6-59.2
3 months	73.0	69.3-76.8
6 months	87.3	83.5-91.1
12 months	92.5	88.7-96.3
18 months	93.4	89.6-97.2
24 months	95.9	92.1-99.7
36 months	97.0	93.2-100.8

**Figure 1:**
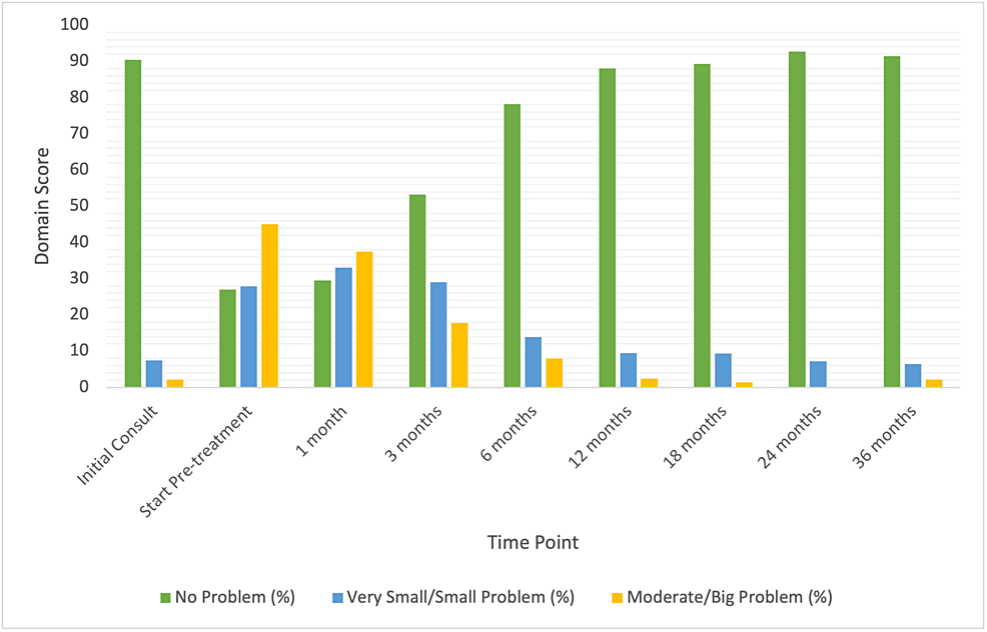
Hot flashes bother following SBRT for prostate cancer by time period Expanded Prostate Cancer Index Composite (EPIC) 13a scores before and after stereotactic body radiotherapy (SBRT) treatment. Patients were stratified into three groups: no problem, very small-small problem, and moderate-big problem.

**Figure 2:**
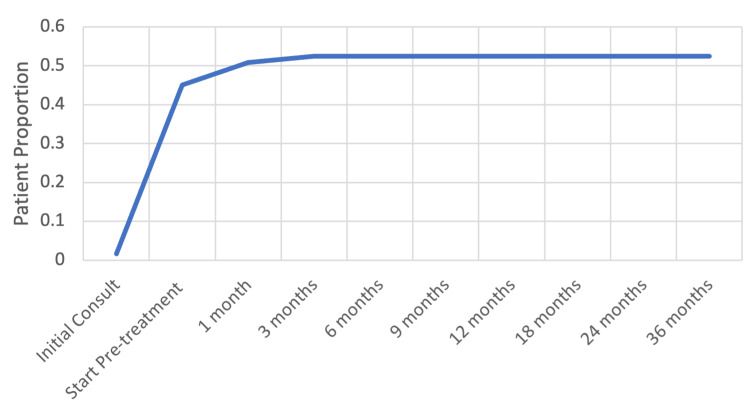
Cumulative incidence of hot flashes before and after SBRT and ADT for prostate cancer ADT - androgen deprivation therapy; SBRT - stereotactic body radiotherapy

Patients reporting a very small/small amounts of bother and moderate/severe bother peaked at one month and pre-treatment (corresponding to receipt of ADT), respectively, and decreased steadily thereafter (Figure 1). High levels of bother occurred closer to ADT receipt before falling to lower levels of bother, then back to baseline levels (Figure 1). The cumulative incidence of hot flashes seen in our patient population was 52.4% for “moderate to big problem” (Figure 2).

**Figure 3:**
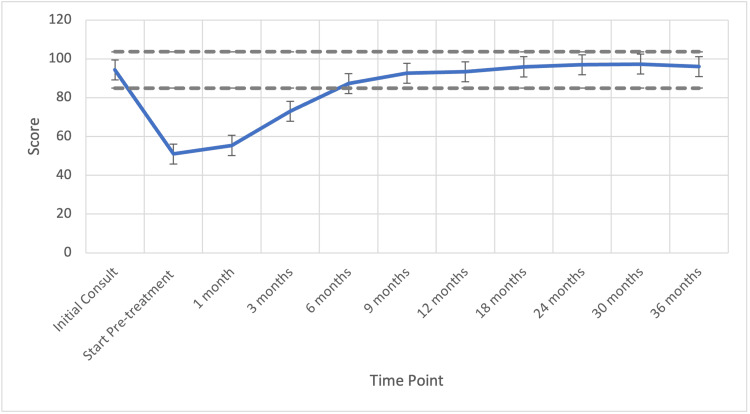
Hot flash reported score over time Gray lines represent MID (Minimally important difference).

**Figure 4:**
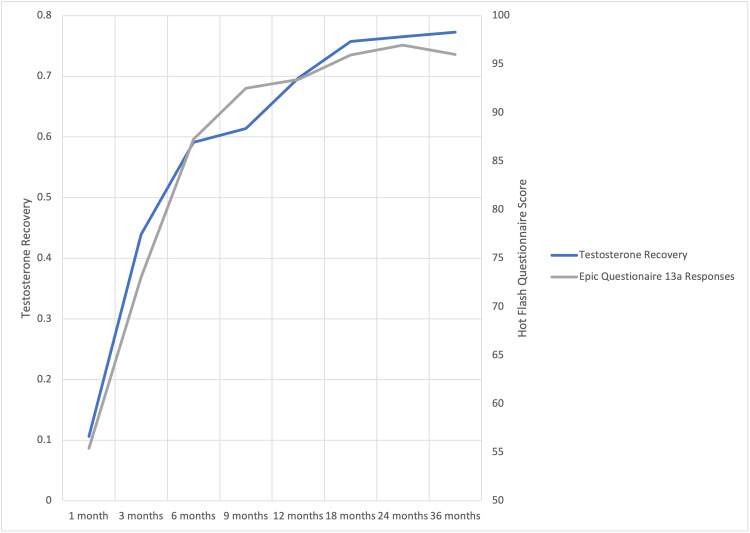
Testosterone and hot flashes over time Testosterone recovery and Epic 13a scores after stereotactic body radiotherapy (SBRT) treatment. Patients were scored from 0 to 100 and testosterone recovery was measured as cumulative incidence across time

The mean time to resolution of clinically and statistically significant hot flashes was six months post-SBRT (MID = 9.51, p<0.01, Figure 3). By six months, testosterone recovered in 60% of patients (Figure 4). There was a strong correlation of 98.6% (R^2=99.7%) between TR and hot flash score, with both values rising and eventually plateauing as time progressed.

## Discussion

Hot flashes are a known complication of ADT, which adversely affects quality of life in men with prostate cancer. While on ADT, 52% of patients reported hot flashes as being a moderate to big problem. Hot flash bother may have significant implications on adherence to treatment recommendations [22]. It is important to communicate with patients about the expected effects of androgen deprivation and discuss the expected recovery with each treatment.

Hot flashes are secondary to the release of hypothalamic catecholamines (i.e., norepinephrine) in response to the decreased LH and FSH [1]. Here, we demonstrate a significant correlation between TR and symptomatic hot flashes. We have previously reported that the median time to recovery of testosterone levels corresponds to approximately four months following ADT cessation [16]. Previous studies have demonstrated TR after short ADT to range from 13 weeks to two years [11,23]. In the present study, we demonstrated that the mean time to resolution of clinically and statistically significant hot flashes was six months post-SBRT (MID = 9.5, p<0.01, Figure 3) corresponding with 60% TR. There is some evidence that shorter-acting GnRH antagonists result in a faster recovery to baseline testosterone levels, which may be an area for further research [24].

Our results add to previous results demonstrating the transient nature of these side effects [6]. The prevalence of hot flashes peaked at the pre-treatment time point corresponding with receipt of ADT. We demonstrate that short-term ADT may contribute to hot flash symptoms for up to six months prior to recovery to baseline. Hot flashes were rare at time points greater than two years post-SBRT. Hormone replacement therapy is contraindicated in this patient population. However, for significant bother, megestrol acetate, venlafaxine, or gabapentin can be trialed [25-27]. Utilizing testosterone levels can further assist in determining a return to an eugonadal state which may coincide with the resolution of hot flashes and improvement in EPIC-26 scores.

Limitations of this study include its retrospective design. Men likely only reported hot flashes to their physician when bothersome to them and/or their partner. Additionally, a portion of our patients were obese and older, which could confound data as they are likely at a lower baseline for testosterone, and less recovered back to a eugonadal state (66%). Likewise, a portion of our patients were Black, which also could contribute as those patients had a higher rate of return to eugonadal state (85%). Further, we did not collect baseline testosterone levels prior to ADT. Hence, the proportion of baseline hypogonadal patients is unknown. The course of ADT given to patients was also variable, and while most received short courses, there may be some further confounding for those with longer courses of ADT (i.e., six months). Men who had hypogonadism at baseline may have had slower TR and more prolonged hot flash systems [15,16].

## Conclusions

Hot flashes are a bothersome self-limiting symptom experienced by a small percentage of men following prostate SBRT. SBRT and short-term ADT are proven to be safe and effective for treating localized prostate cancer. Hot flashes and testosterone are closely associated with one another, with TR coinciding with a resolution of hot flashes. Reassurance of the short duration of hot flashes may assist in reducing patient anxiety. Hot flashes followed the TR trend closely, and follow-up monitoring of testosterone levels may allow for guidance to limit bother of these temporary changes.
